# Knowledge, safety, and teamwork: a qualitative study on the experiences of anaesthesiologists and nurse anaesthetists working in the preanaesthesia assessment clinic

**DOI:** 10.1186/s12871-022-01852-w

**Published:** 2022-10-03

**Authors:** Eirunn Wallevik Kristoffersen, Anne Opsal, Tor Oddbjørn Tveit, Mariann Fossum

**Affiliations:** 1 Department of Health and Nursing Science, University of Agder, Grimstad, Kristiansand, Norway; 2 Department of Anaesthesiology & Intensive Care, Sørlandet Hospital, Kristiansand, Norway; 3 Department of Clinical Research, Sørlandet Hospital, Kristiansand, Norway; 4 Department of Technology and E-Health, Sørlandet Hospital, Kristiansand, Norway

**Keywords:** Anaesthesia, Preoperative care, Outpatient clinics, Experience, Qualitative research, Patient safety, Nurses, Anaesthesiologist, Patient participation

## Abstract

**Background:**

The preanaesthesia assessment clinic (PAC) has been shown to contribute to safe anaesthesia assessment in hospitals. In the PAC, patients are assessed with an interview and can also ask relevant questions about anaesthesia. The intention is to ensure that patients are comprehensively prepared for the surgery and hospital stay. Although earlier studies have assessed the effects of PAC, attitudes and satisfaction of the healthcare personnel working in PAC remain unknown. Thus, this study aimed to examine the experiences of anaesthesiologists and nurse anaesthetists working in PACs as well as to explore barriers and facilitators in this context.

**Methods:**

A descriptive qualitative approach was used to explore the experiences of anaesthesiologists and nurse anaesthetists working in PACs. Thirteen semi-structured interviews were conducted using face-to-face, telephone, or digital platforms in five hospitals in west, south, and north Norway between 2020 and 2021. The interviews were transcribed and thematically analysed according to Braun and Clarke’s six-steps semantic reflexive analysis.

**Results:**

Four themes and nine subthemes were identified through an active analysis process, including developing competence in clinical anaesthesia practice, identifying barriers and facilitators influencing collaboration and teamwork, improving patient safety and outcomes through structured assessment, and identifying other organisational factors affecting the delivery of healthcare to surgical patients.

**Conclusions:**

Working in the PAC contributed to competence development among the personnel. Additionally, teamwork was considered important for the proper functioning of the PAC. Patient safety was perceived as improved owning to the structured assessment in PAC, with patients getting the opportunity to improve their knowledge and being more involved in the upcoming anaesthesia.

**Supplementary Information:**

The online version contains supplementary material available at 10.1186/s12871-022-01852-w.

## Background

According to the international standards for safe practice of anaesthesia developed by the World Health Organization and World Federation of Societies of Anaesthesiologists, every patient should be cared for at the highest standard of safety possible, independent of whether the provider is an anaesthesiologist or non-anaesthesiologist [1]. New challenges are emerging in the field of anaesthesia, like the growing population of elderly patients with complex diseases [2] and the increased need for the complexity of surgical procedures. Preanaesthesia assessment clinics (PACs) have been developed as a service unit to surgeons, anaesthesia personnel, and individual patient needs [3]. The main goal is to identify, communicate, and minimize the patient’s specific risk of anaesthesia and surgery. The patient is informed and engaged in decisions [4]. Research in this field may be challenging as PACs are organised differently in different hospitals to suit patients’ needs and the structure of the individual hospital [5].

In Norway, all hospitals follow the guidelines of the Norwegian standard for the safe practice of anaesthesia. Inspection and preoperative information documentation is carried out by a nurse anaesthetist or anaesthesiologist [6]. Every patient is classified using the American Society of Anesthesiologists (ASA) physical status classification system [7]. Additionally, medical information, including height and weight, previous anaesthesia, allergies, current medication, bleeding disorders/coagulation status, physical and mental state of health, airways and intubation, results of any relevant supplementary examinations, preoperative pain and fasting, and a plan for postoperative care, including pain management must be taken into consideration and if necessary, checked and cleared by the anaesthesiologist before administration of anaesthesia [6, 8]. Not all hospitals in Norway have a PAC for different organisational reasons.

Amaya et al. reported that most anaesthesiologists working in a hospital without a PAC responded that they were unable to set up a PAC despite admittedly needing one. The major reason was a shortage of human recourses [3]. Lemmens et al. found that satisfaction with the current organisation of preoperative evaluation was highest among respondents of hospitals with a complete PAC and lowest among respondents without a PAC. Improved logistics in the preoperative pathway was mentioned most frequently as a benefit of the PAC [9]. Furthermore, studies on the attitudes and satisfaction of the health care personnel working in the PAC is needed [10] and the contributions of PACs. The information obtained from the PAC personnel might clarify the unanswered questions in the field of anaesthesia, yield new information, and contribute to further research.

## Methods

### Aim

The aim was to gain an understanding of the experiences of anaesthesiologists and nurse anaesthetists in the PAC, including context-specific barriers and facilitators.

### Design

This study used a qualitative descriptive design to gain an understanding of the PAC as a complex phenomenon [11, 12]. Semi-structured interviews were used to identify the experiences and perspectives of anaesthesia personnel [11]. Data were analysed according to Braun and Clark’s semantic reflexive thematic analysis, constructing themes and subthemes to summarise how the participants depict their experiences [13, 14]. This is a relevant methodological tool for research in anaesthesia and for understanding experiences of healthcare personnel [15].

### Sample and context

The participants consisted of four anaesthesiologists and nine nurse anaesthetists who were working or had previously worked at a PAC. They were employed at various hospitals in Norway, including one regional hospital, one university hospital, and the rest were large emergency hospitals. The term regional hospital is used when a hospital is designated as the main hospital in a region and offers regional and national medical services, while a university hospital is linked to a university and participates in the teaching of medical students and clinical research. The term large emergency hospital is used for a hospital with a catchment area of more than 60–80,000 inhabitants, which offers a wide range of emergency services, including emergency surgery and several medical specialities [16]. One hospital was in the southern part, two in the western part, and two in the northern part of Norway. The included hospitals were from three of four health trusts in Norway. We chose to include both nurse anaesthetists and anaesthesiologists because of their close collaboration in the anaesthetic wards [6].

The study context was based on the following inclusion and exclusion criteria presented in Table 1.

**Table 1:** Inclusion and exclusion criteria

Inclusion criteria	Exclusion criteria
Public hospitals in Norway with emergency status and an established PAC Nurse anaesthetists or anaesthesiologists with former and/or present experiences from a PAC No time limit or number of cases that the participants had assessed	Hospitals without a PAC Hospitals with only the anaesthesiologists assessing the patients at the PAC

*PAC* Preanaesthesia assessment clinic

The main author telephoned public hospitals in Norway with emergency status to find out if they had established a PAC and how it was organised. Our target was to contact all public hospitals in Norway by telephone, but it was challenging to reach managers at all hospitals to get information about their organisational structure. Thus, we stopped when we had reached 38 hospitals. Twelve of these 38 hospitals had both nurse anaesthetists and anaesthesiologists working together and requests were sent to five random hospitals with experiences from PACs. The main author mailed information letters and consent forms to managers of anaesthesia departments who organised the recruitment. The department manager or professional development nurse in the hospital’s anaesthesia department oversaw the recruitment of the study participants to ensure voluntary participation. In addition, two participants were recruited as a result of snowball sampling. The main author sent these forms directly to participants who were recruited with snowball sampling.

The anaesthesia personnel worked either permanently in the anaesthesia department with one to three days per week in the PAC or a fixed percentage up to 50% of their time at the PAC. Their engagement in PAC varied owning to variation in available personnel. The hospitals had electronic healthcare record systems, except for one that still used a paper-based record system. The PACs had been implemented from 3 to 24 years and collaborated with different surgical wards. The timing of the preanaesthesia assessment varied from six weeks to one day before the patients were admitted for surgery. Time spent on each assessment performed at the PAC varied from 10 min up to 1 h depending on the type of surgery, ASA classification, and if something unexpectedly happened that could influence the anaesthesia and needed more investigation, and if the patient needed more information. Table 2 shows the characteristics of participants.

**Table 2:** Demographic characteristics of interviewed nurse anaesthetists and anaesthesiologists (*N* = 13)

**Characteristics**	**Number (%)**
Sex	
Female	9 (69.2)
Male	4 (30.8)
Age	
31–40 years	1 (7.7)
41–50 years	4 (30.8)
51–60 years	5 (38.4)
61–65 years	3 (23.1)
Position size	
50–75%	1 (7.7)
76–99%	1 (7.7)
100%	11 (84.6)
Number of years of working as registered anaesthesia personnel	
2–10 years	4 (30.8)
11–20 years	5 (38.4)
21–30 years	1 (7.7)
31–40 years	3 (23.1)
Number of years working in the PAC	
Less than 1 year	1 (7.7)
1–4 years	7 (53.8)
5–8 years	4 (30.8)
9 and more years	1 (7.7)

*PAC* Preanaesthesia assessment clinic

### Data collection

A semi-structured interview guide was developed collaboratively by the research team. A pilot face-to-face interview was conducted with a nurse anaesthetist working in a PAC to determine if the interview guide contained relevant questions, leading to minor changes in the interview guide. In the beginning of the interview, some questions about the background characteristics of the participants were asked, followed by questions on the reasons why their hospital implemented a PAC. Further, we inquired about their experiences working in the PAC, how the work was organised, and what feedback was received from the patients who were assessed in the PAC. To get the informants to elaborate on their experiences, we used words, such as: ‘Tell, describe, what did you experience, what do you think, how did you perceive, and what happened then?’.

In one hospital, the first author met the participants face-to-face. Since the coronavirus disease (COVID-19) pandemic made it impossible to travel, a digital platform was used for one interview and attempted in two interviews. However, because of firewall and poor internet issues, it was changed to telephone with speaker, and this was used for the rest of the interviews. An audio tape recorder without internet connection was used for all interviews. Only the researcher was present together with the participant in the room during the interview. However, in one telephone interview, a colleague was in the same room as the participant.

The interviews were conducted in spring of 2020. However, one interview was completed in January 2021 because of a delay in the project. All participants talked eagerly about the topic and the interviews lasted from approximately 45 min up to 1 h. By the tenth interview, we realised that we had relevant and adequate data that mesh with the aim of the study; thus, we conducted three more interviews before stopping recruitment [13, 17].

The first author (EWK) transcribed six of the interviews, while seven were transcribed by a professional transcriptionist verbatim in Norwegian because of time constraints in the project. A thorough orthographic transcription was done that captured what was said. To add, some basic details of how things were said, such as notable pauses in speech and filler words, were added and emotional details, such as laughter and yawning, were included [18]. As the transcriptionist was not an anaesthesia personnel, the first author (EWK) listened and corrected the transcribed text to improve the anaesthesia phrases and words.

### Ethical considerations

Approval was obtained from the hospital’s research department, faculty’s research ethic committee, and Norwegian Centre for Research Data (NSD; project number 61318) [19]. Participants received verbal and written information about the study and were explained about the possibility to withdraw at any time without any consequences. Further, they received a guarantee on confidentiality and were assured that only the research team would have access to the collected data. The audio recordings were deleted after completion of transcription and analysis. The study was carried out in accordance to the World Medical Association declaration of ethics for medical research [20].

### Data analysis

An inductive, semantic reflexive thematic analysis, according to Braun and Clarke, was performed on the transcribed interviews and uploaded to NVivo 12 for data management [21].

In the first phase of the thematic analysis, we familiarised ourselves with the dataset by listening to the audio data, transcribing, reading, rereading, and adding some notes to grasp the meaning of the text. The main author read all the text and all authors read parts of the text to ensure rigour in the analyses phase. In the second phase, we identified segments of data that were relevant and meaningful for our aim. These coding steps summarised and reduced the content and captured the ‘analytic take’ on the whole data set. Code labels were created as nodes in NVivo and relevant segments of the data were compiled for each code [13]. We tried to keep the codes close to the meaning of the text so that we could easily understand the content. This approach is named semantic according to Braun and Clarke [13]. Coding of the transcripts was completed by the first author and checked by all authors. In phase three, we started to identify the character of the potential themes and subthemes by clustering together the codes that shared a central idea or concept and could answer our aim of the study [11, 13, 22]. The writing process was also started here because it was easier to see the connection between the codes when writing it down. The development of themes was conducted in an active process involving all researchers based on the data, aim, our knowledge, and insights. Codes usually captured a specific meaning, themes described a broader and shared meanings [13]. In phase four, we went back and forth checking the themes and subthemes in relation to both the coded text as well as the full data set. Radical changes were required several times and we tried to stay as reflexive and close to the analysis method as possible. In phase five, we focused our analysis, and some changes were again required to make sure that each theme was clearly defined. Finally in phase six, we finished the writing process to compose a coherent and persuasive story about the dataset that answered our aim [13, 22]. The main author had some former experience with qualitative research and all authors worked closely together to ensure that every step was taken to maintain the trustworthiness of the study. Examples from the analysis process are presented in more detail (see Additional file 1).

## Results

Four themes and nine subthemes were obtained from the analysis and are presented in Table 3.

**Table 3:** Themes and subthemes

Developing competence in clinical anaesthesia practice	Barriers and facilitators of collaboration and teamwork	Improving patient safety and outcomes through a structured assessments	Organisational factors affecting anaesthesia personnel and delivery of healthcare to surgical patients
Increased learning through the structured assessment of patients Nurse anaesthetists increased responsibility when working in the PAC	Internal teamwork Interdisciplinary collaboration	Improving patient knowledge, experience, and involvement Perceived improvement of patient outcomes	Effects of having a PAC Time factors Increase in workload pressure

*PAC* Preanesthesia assessment clinic

### Developing competence in clinical anaesthesia practice

The PACs were experienced as an arena for learning more about anaesthesia, and the participants perceived an increase in their competence through working in the PAC.

‘For my own part, I learn a lot. The fact that you must complete it (anaesthesia form) yourself, I think it is very instructive to find out what is important to obtain good quality information’ (Participant 11).

Nurse anaesthetists expressed that they understood the anaesthesiologist’s job better when they worked in the PAC, making it easier for them to understand the arguments provided after patient assessment. A common theme for the anaesthesia personnel was that they learned a lot about different medications, which they considered important knowledge. An anaesthesiologist reported experiencing a rapid development in different type of medications with missing national guidelines on some type of medications in relation to surgery.

‘So, I miss some updated national guidelines on regular medications in relation to surgery’ (Participant 7).

Anaesthesiologists pointed out that anaesthetic nurses learned from assessing ASA 1 and 2 patients, but it was also important that they participated in assessing the ASA 3 and 4 patients owning to the learning experience.

‘It is important that they do not miss out on this learning arena if all the difficult ones (patients) are run over to the anaesthesiologist automatically’ (Participant 8).

Nurse anaesthetists were also recognised from the anaesthesiologists for being good at talking to the patients and their notes were well written in the medical records. However, the anaesthesiologists fronted that the assessments done by their own profession were more thorough and more professionally conducted. On the other hand, the nurse anaesthetists said they informed the patients more practically than the anaesthesiologists. In addition, nurse anaesthetists thought that their work at the PAC reflected their competence and that they would achieve a higher professional status. Participants also said that with time, they gained experience from their work, making it easier to perform their duties.

‘You must get used to the work, learn how to work efficiently and smoothly with the system that is used in the PAC. And when you gain control and feel that you have mastered it, then I really think it’s a very decent way to work’ (Participant 1).

Nurse anaesthetists described that working in a PAC involved a lot of responsibility. They encountered different settings compared to what they were used to in the anaesthesia wards. They signed the documents and were more responsible for their own assessments of the patients. Additionally, they were afraid of making mistakes or forgetting something important.

‘In the past, it was the anaesthesiologist’s responsibility to perform the assessment on the patient preoperatively either in the ward or only on paper. So, when I am in charge of the assessment, I feel very responsible. I am very scared of not catching things that I should have’ (Participant 3).

The anaesthesiologists demonstrated that the overall responsibility lies within the anaesthesiologist in charge of the PAC that day and that there were guidelines to follow. However, the anaesthesiologist emphasised that there were more undefined responsibility assignments between the surgeon and anaesthesiologists regarding the PAC.

### Barriers and facilitators of collaboration and teamwork

Anaesthesiologists and nurse anaesthetists explained that they were depending on each other to ensure that the PAC functioned properly. Some said that they got to know each other better, not only as professionals, but also on a personal level.

‘There will always be a system component and personal component. It will be a bit variable between the nurses and doctors that are working on a given day’ (Participant 7).

Overall, the teamwork was perceived as well functioned, because they all saw the advantages of having a PAC. However, sometimes, the anaesthesiologists were needed elsewhere/had other assignments and the nurse anaesthetists had to call another anaesthesiologist for help. This was experienced as difficult by nurse anaesthetists. However, there were some anaesthesiologists that did not like working in the PAC, and hence, the teamwork did not function properly. Even so, some anaesthesiologists pointed out that it made sense to them that they should collaborate with nurse anaesthetists in the PAC, because they worked so closely in the OR.

‘It gives a new arena of collaboration with the nurse anaesthetists, which I think is positive’ (Participant 8).

Several participants said that they missed feedback from their colleagues working in the anaesthesia ward (both nurse anaesthetists and anaesthesiologists). However, some participants said that they had only experienced positive feedback, and especially from the anaesthesiologist on whom nurse anaesthetists rely for their daily work.

The PAC was experienced as a place where collaboration between surgeons, wards, nurses, and interns was important during daily procedures to achieve the best outcomes for the patients even though they all have a different focus.

‘I think it has been a good arena to collaborate (teamwork) with the surgeons. One is almost forced to have more contact with each other, and I think it has been very positive’ (Participant 10).

However, nurse anaesthetists and anaesthesiologists said that they sometimes had to make sure that surgeons did their job. For instant, if they did not remind them to stop the medications preliminarily, they knew that the planned surgery would be postponed. They also pointed out that sometimes the surgeon did not understand their work and focus.

‘I used to refer to Lorentz Gran, the master of anaesthesia in Norway. He starts the textbook by saying that there are small and large procedures in surgery, but not small and large anaesthetics. And I think we find that it is not always the surgeons who thinks about these consequences’ (Participant 6).

### Improving patient safety and outcomes through a structured assessment

The conversations between the patients and anaesthesia personnel in the PACs were emphasised as important and how the information given to the patients could influence the patient’s feeling of safety.

‘We had an adult lady with intellectual disabilities who needed surgery. She told us she was afraid of people in green clothes. We then asked her if it was better if we used blue clothes. She liked blue, so it went very well’ (Participant 6).

A common theme was that the PACs provide an excellent opportunity for patients to ask questions, obtain information about anaesthesia, correct misunderstandings, and talk about former anaesthesia experiences.

‘There are a lot of patients who say that they were not afraid of the surgery, but they were afraid of never waking up again. I think it is very nice that we can inform them about what anaesthesia in the 20th century means and how it works. I think it is an advantage for the patients to get this information’ (Participant 6).

Further, the participants expressed that a PAC gave the patients an opportunity to be a part of the decision-making process of the anaesthesia to come. However, the safest way to have anaesthesia was always prioritised.

‘And then they tell us about previous experiences, both good and bad. And then we try to find something that fits them. So, they can present their own views’ (Participant 9).

Anaesthesia personnel talked about the advantages of having a PAC related to patient safety and felt that it was a lot easier to identify patients at risk when they had time to sit down, and physically see and talk to the patients with the journal and self-declaration form in front of them before surgery. Some potential complications could be discovered just by looking at the patient’s neck, mouth, hands, and body.

‘You absolutely identify the risk factors. There are many things like allergies for instance, it does not have to be in the journal. Then it sort of comes in a subordinate sentence from the patient. Yes, I had a terrible reaction to penicillin once. You know, things like that can just pop-up when we interview them. So, you may get information that has not been noted in the journal’ (Participant 13).

The participants said that their experience was that if the anaesthesia assessment was done on the day of the surgery, the patient could more easily forget to inform them about certain conditions because their focus was elsewhere. Some cases needed to be referred to other specialties for further examination before it was safe to perform the surgery, thus avoiding cancellations on the day of surgery or an unwanted incident in the operating room (OR).

‘Because you manage to identify those who may need cardiological supervision, those who must have it before we can operate on the patients. Then, they do not have to be cancelled on the day of surgery. So absolutely, I think that this clinic is a win’ (Participant 13).

Anaesthesia personnel stated that the implementation of a PAC had led to the development of protocols that improved the follow-up of premedication and identification of pain and nausea, which was described as positive.

‘One of the advantages of a PAC is that almost everyone is prescribed a premedication, which has a lot of impact postoperatively. In relation to many patients coming from the wards or emergency patients who have not received premedication, they obviously have a lot more pain postoperatively’ (Participant 3).

According to participants, the records of all patients assessed in the PAC contributed to quality assurance. Anaesthesia personnel working in the anaesthesia wards used these records to obtain information about the patient on the day of surgery (together with previous patient records and blood tests), and it gave them a quick overview of the most important data that were important for anaesthesia.

‘Maybe the patient has been in the hospital 10 times in the last two years, so you could scroll back the previous patient records, but you are unsure if you can capture everything; however, here you have a record that captures the essence. I think it is obvious, if I have a patient who has been to the PAC, then I learn a lot from that record and I use it a lot’ (Participant 2).

Further, this patient records both saved time on the day of surgery and if the same patient came back for surgery on another occasion.

‘We get good feedback from those who read the patient records from the PAC. It makes the everyday life in the OR easier as I can quickly get an overview of the patient’s health’ (Participant 5).

### Organisational factors affecting anaesthesia personnel and delivery of healthcare to surgical patients

The anaesthesia personnel emphasised the workflow with the surgical patients was streamlined with the use of PACs, resulting in an increased efficiency of surgery.

‘Yes, it was probably to get a better patient flow, to avoid situations where the patients were not well prepared when they came to the OR. They could get the assessment they needed in advance and there would be less cancellations of surgery because of poor preoperative assessment. Additionally, it probably helped to organise us a little better as well’ (Participant 5).

Anaesthesia personnel had different opinions on what kind of patients should be assessed at the PACs, and the organisational structure regarding patient groups differed among the hospitals. Some hospitals referred all patients to the PAC, including children, while others referred only patients of specific surgical and orthopaedic specialties. Some participants said that the available resources decided what kind of patients could be seen in the PAC.

‘Then there are some who think that it has less to do with healthy patients, that if the self-declaration is unproblematic then there is little point in calling them in. But at the same time, for example people can give a very bad self-declaration on potentially difficult airways’ (Participant 7).

Participants expressed that the PAC reduced the length of hospital stay, but no numbers were provided. They said that everything was prepared in advance; consequently, the patients did not need to be admitted before the day of surgery.

‘The PAC is part of a reduced length of hospital stay. Instead of coming the day before, and going through this the day before, most people are now.... cleared in advance. The vast majority can also come on the same day as the operation’ (Participant 1).

Participants said that they experienced that cancellations of surgery and delays in the OR were reduced when patients attended the PAC. Therefore, the hospital could utilise the operating capacity and fit in another elective surgery patient. Participants mentioned that this must be financially advantageous for the hospitals.

‘Different surgical specialties said they got more patients through because they attended the PAC, and they could avoid cancellations of surgery because of lack of correct preparations’ (Participant 4).

To have enough resources and time scheduled for each patient was an important aspect to make the work situation optimal. It is difficult to know in advance what kind of patients were coming in regarding the health status, medical history, and planned surgical procedure.

‘The time required is not easy to predict; therefore, we need to understand that some patients need more time than the others. There must be some possibilities to make room for demanding patients that need more time’ (Participant 1).

How participants experience the workload varied, partly because of the different organisational structure of the hospitals. Some of the working days could be busy, stressful, and tiring.

‘It is sometimes so tiring that one dreads the day on the PAC, because you sometimes have up to 16 patients’ (Participant 13).

Others mentioned that the days in the PAC could be slow and gave them energy for the rest of the week when they experienced busy working days at the anaesthesia ward. They wished that days at the PAC could be more predictable. However, some participants highlighted that working in the PAC was a good change from other days when they were working in the anaesthesia departments. Nevertheless, most participants said that they would not work at the PAC fulltime, while others considered working there as a duty because they saw how important the PAC was for anaesthesia patients and anaesthesia departments. Participants also pointed out that a lot of resources were spent on the PAC, but they were worth it.

‘It was not like I was cheering when I got that job, and when people are put in the PAC, it is often with some reluctance because it is not something they perceive as fun work, but most people realise that it is useful work’ (Participant 7).

The COVID-19 pandemic stopped many activities in the PAC, and some participants felt that these activities did not necessarily have to be performed in a physical room in the hospital, but could perhaps be completed on a digital platform. For patients travelling long distances, they suggested that this could be a better solution.

## Discussion

This study sought to examine the experiences of anaesthesia personnel working in PACs, and to explore the barriers and facilitators of the PAC implemented in hospital settings. Although working in the PAC was perceived to contribute to increased competence by nurse anaesthetists, it was also associated with considerable responsibility. Anaesthesia personnel were depending on each other to make the PAC function properly, and therefore, teamwork was an essential element of the PAC. Participants perceived that the PAC contributed to improved patient safety and empowered the patients. In addition, time pressure and high workload were reported although there was a variation in the workload.

A common theme in this study was that competence improved for the anaesthesia personnel when working in PACs. Safe practice in anaesthesia requires a high level of competence and expertise in medical diagnosis, pharmacology, physiology, anatomy, and considerable practical skills [1]. Even if it is a long journey from a novice to an expert in this field, development of competence contributes to higher satisfaction amongst workers and prevents personnel turnover and burnout [23] and contribute to a reduced number of sick leaves for the anaesthesia departments [24]. When a clinical role is stimulating, challenging, and meaningful, it is a strong predictor of job satisfaction among both anaesthesiologists and nurse anaesthetists [25]. For the anaesthesia department this may contribute to improvement in the clinical performance and productivity of the anaesthesia personnel. However, high workload may again contribute to burnout [24].

Although it was decided that nurse anaesthetists assessed ASA 1 and 2 patients, patients were often sicker than anticipated. Nevertheless, often the anaesthesiologist’s quality assessment assured the nurse anaesthetists`s assessment at the end of the day. However, a study proposed that nurse-led preoperative assessment can be useful in identifying and defining patients` risk factors and not just for surgery, but for the entire perioperative care trajectory [26]. Furthermore, anaesthesiologists stated that it was important for nurse anaesthetists to participate in the assessment of higher ASA patients because it can lead to improved learning outcomes. A study that evaluated registered nurses and the reasons why they stayed in work found a positive relationship between perception, emotions, and work motivation. This also affects work performance and potential to solve complex work-related problems in a positive manner [27]. The complexity of anaesthesia care is more cared for with highly motivated workers in the anaesthesia department. A qualitative study performed on nurse anaesthetists and OR nurses revealed that the feeling of being replaceable and not important in the workplace was a reason for quitting their job [28]. Ahlstedt et al. found evidence supporting the results of our study that positive work motivation among registered nurses was to work independently in a friendly atmosphere with colleagues from the same profession and in trusting collaboration with physicians in addition to receiving feedback for their work itself, integrated with learning [27]. However, in our study, the participants reported mixed feedback from colleagues regarding their work in the PAC.

One interesting finding from this study is the importance of team collaboration in the PAC, particularly the opportunity to discuss cases and get to know each other better. These findings reflect those of a study by Karlsson et al., in which registered nurses highlighted the importance of team collaboration and communication for ensuring patient safety. Nurses valued working with colleagues they knew and could rely upon for support. This was a positive work factor, which contributed to their intention to continue working in the current workplace [23]. Other studies have also reported that social ties can influence teamwork [29] and patient satisfaction [30]. When strong social ties were present in the workplace, the hierarchy is flattened [29]. This may contribute to improved teamwork in the OR, where the emphasis of efficiency is high, and helps prevent mistakes that could harm surgery patients. Additionally, interdisciplinary teamwork in the PAC involving the surgeons, wards, nurses, and interns was reported as important in our study. Participants had to collaborate to achieve the best patient outcomes, even if they had a different professional emphasis. However, participants sometimes felt that surgeons did not understand their work assignments. Park et al. investigated the satisfaction of the surgeons with the PAC and found it difficult to state the tendency of surgeons’ perception of the PAC [10].

Another interesting finding in this study was that the participants felt that the PAC was a major contributor to patient safety in many areas in the fields of anaesthesia and surgery. Safety culture is a complex phenomenon in healthcare and a subset of organisational culture. This means that members of an organisation have a shared set of values, beliefs, and patterns of behaviour regarding patient safety [31]. It was mentioned by anaesthesia personnel in this study that a PAC ensures that the patients get the best start for anaesthesia. After conversations with patients and reading their former records, participants could discover elements that were crucial for the upcoming anaesthesia, such as unknown diagnoses, and former and possible anaesthetic complications. Harries et al. found that patients did not notify medical professionals about important medical information because either they thought it was not important or forgot to tell [32]. Moreover, a study reported that nearly four in 10 respondents faced difficulties in accessing, understanding, appraising, and applying health information [33]. This is supportive of our findings where the participants embraced the use of a PAC as they could focus on the patient’s needs and ensure that the patient had understood what they needed to do before the surgery, thus avoiding cancellations or interoperative events in the OR. According to the ASA, it is important to undertake a structured assessment of the patient preoperatively to avoid unexpected incidents in the OR [7]. It is essential that personnel be familiar with the guidelines and standards of preoperative evaluation to accurately stratify patient risk and advocate for patient safety [34].

Furthermore, in this study, participants highlighted the importance of talking with patients. It is common for surgery patients to experience fear and anxiety before anaesthesia [35]. For example, a study found that patients in the preoperative phase might have a fear of obscurity, being in a strange situation, and feeling of loss of control [36]. Preoperative anxiety may lead to both physiological and psychological responses, which may cause problems for both the surgery and anaesthesia [35, 37]. Participants in a qualitative study commented that educating patients through perioperative dialogue gives patients the opportunity to manage their fears, resulting in better patient participation and well-being [38]. This is consistent with the findings in our study where participants said that a conversation contributes to a good dialogue between anaesthesia personnel and patient, leading to a feeling of safety and less misunderstandings. Patient empowerment through an information booklet and diary-keeping has been reported to improve the quality of care in elderly cancer patients with postoperative pain [39].

Participants in this study stated that because of the PAC, less time was spent in the OR on the day of surgery, which could be a benefit for the hospitals using their personnel more efficiently. The rising demand for surgery in Norwegian hospitals [40] has created a constant pressure from the political class, hospital leadership, patients, and relatives. Consequently, it is essential to improve the patient flow and optimise surgical management to provide timely treatments to patients and maximise the utilisation of the available resources [41]. Anaesthesia personnel in this study could focus more on the well-being of patients in the PAC, and they reported less unexpected events in the OR, resulting in a reduction in cancellations of surgery. An integrative review pointed out that cancellations of surgery are a significant quality issue in health care and are linked to the undesired outcomes of waste of resources, patient dissatisfaction, and increased health costs. Most causes of cancellations of surgery are preventable [42]. However, some participants in our study commented that they always felt they had to work faster, and the PAC contributed to this. Sanfilippo et al. investigated factors associated with burnout in anaesthesia personnel, and high workload was one contributing factor [43]. Therefore, it is important to emphasise that the results of this study are relevant to healthcare services, focusing on organisation, well-being, and safety of patients. This way of organising preoperative care could be an inspiration for hospitals not having these services. However, more research on the differences between the nurse anaesthetists, anaesthesiologist, hospitals, and efficiency of a PAC are needed as a contribution to the field.

### Strengths and limitations of the present study

The skewed distribution between the number of anaesthesiologists, nurse anaesthetists and sex is a limitation of this study. Voluntary participation might be the main reason for this. An increase in the sample size could influence the heterogeneity of the participants and our results. However, the participants were eager to talk about the PAC and provide a unique insight of their experiences. Malterud et al., claims that the more information the sample holds, the lower number of participants is needed [16]. In addition, the strength and rigour of this study is the number of different hospitals participating and their diverse locations. A digital platform and telephone were used because of the limited opportunities to travel and conduct face-to-face interviews due to the COVID-19 pandemic. However, we were aware that telephone interviews could lead to reduced information quality compared to face-to-face encounters, because of the limited insight into the respondent’s body language. Therefore, we might have missed the opportunity to ask follow-up questions based on the expressions of the participants. However, telephone interview may provide a calmer and more private setting and yield valuable information [44] as this study shows. Another limitation might be that the interviews were conducted by only one person. Transcripts were not returned to participants for participant validation [13]. The use of focused group interviews was considered instead of individual interviews to encourage interaction and discussion with other participants in the group to bring forward new information [12]. However, recruiting several participants during work hours or in their spare time was considered a major obstacle. Furthermore, individual perspectives may be less effectively explored due to reluctance of the group members to speak frankly in front of others [11]. Some of the quality criteria suggested by Braun and Clarke such as transparency considering the research process and the researchers`s role could possibly have been even more achieved revaluating the result according to a more stringent qualitative approach [13]. But to ensure trustworthiness of this study, all stages of the research process, including data collection and data analysis are detailed described. The results may therefore be transferable to other hospitals including a PAC around the world despite being performed in the Norwegian hospital settings.

## Conclusions

This study is one of the first studies to explore the experiences of the anaesthesiologists and nurse anaesthetists working in a PAC. The PAC was perceived as contributing to competence development amongst anaesthesia personnel, and teamwork was reported as an important factor to ensure that the PAC ran smoothly. Further, participants pointed out that a PAC led to better opportunities for patients to be involved in decision-making, in addition to improving patient safety and outcomes through a structured assessment. Overall, the participants expressed that the PAC contributes to several advantages for the anaesthesia personnel, patients, and anaesthesia departments.

## Supplementary Information

**Additional file 1.** Examples from the analysis process when generating themes.

## Data Availability

The transcribed interviews and data analysis in NVivo12 during the current study are available from the corresponding author on reasonable request.

## Abbreviations

ASA: American Society of Anesthesiologists
OR: Operating room
PAC: Preanaesthesia assessment clinic
COVID-19: Coronavirus disease

## References

[1] Gelb AW, Morriss WW, Johnson W, Merry AF, Abayadeera A, Belîi N (2018). World Health Organization-World Federation of Societies of Anaesthesiologists (WHO-WFSA) international standards for a safe practice of anesthesia. Anesth Analg.

[2] Peden CJ, Campbell M, Aggarwal G (2017). Quality, safety, and outcomes in anaesthesia: what's to be done? An international perspective. Br J Anaesth.

[3] Amaya F, Shimamoto S, Matsuda M, Kageyama K, Sawa T (2015). Preoperative anesthesia clinic in Japan: a nationwide survey of the current practice of preoperative anesthesia assessment. J Anesth.

[4] Tariq H, Ahmed R, Kulkarni S, Hanif S, Toolsie O, Abbas H (2016). Development, functioning, and effectiveness of a preoperative risk assessment clinic. Health Serv Insights.

[5] Bader AM, Sweitzer B, Kumar A (2009). Nuts and bolts of preoperative clinics: the view from three institutions. Cleve Clin J Med.

[6] Ringvold EM, Bekkevold M, Bruun AG, Børke WB, Finjarn TJ, Haugen AS (2018). Norwegian standard for the safe practice of anaesthesia. Acta Anaesthesiol Scand.

[7] Committee on Economics (2020). ASA physical status classification system: - American society of anesthesiologist.

[8] The Norwegian Association of Nurse Anesthetist. Guidelines for the practice of anesthesia in Norway. ALNSF; 2020. Available from: https://www.alnsf.no. Accessed 01 Jan 2022.

[9] Lemmens LC, Kerkkamp HE, van Klei WA, Klazinga NS, Rutten CL, van Linge RH (2008). Implementation of outpatient preoperative evaluation clinics: facilitating and limiting factors. Br J Anaesth.

[10] Park JY, Park SJ, Ri HS, Choi EJ, Choi YM, Yoon JU (2019). Experience of operating an anesthesia preoperative evaluation clinic in South Korea: an observational study of surgeons' satisfaction. Medicine (Baltimore).

[11] Braun V, Clarke V (2014). What can “thematic analysis” offer health and wellbeing researchers?. Int J Qual Stud Health Well-being.

[12] Polit DF, Beck CT. Essentials of nursing research: appraising evidence for nursing practice. 8th ed., International ed. ed. Philadelphia: Wolters Kluwer/Lippincott Williams & Wilkins; 2014.

[13] Braun V, Clarke V. Thematic analysis: a practical guide. Los Angeles: Sage Publications Ltd; 2021.

[14] Braun V, Clarke V (2021). One size fits all? What counts as quality practice in (reflexive) thematic analysis?. Qual Res Psychol.

[15] Jowsey T, Deng C, Weller J (2021). General-purpose thematic analysis: a useful qualitative method for anaesthesia research. BJA Educ.

[16] Norwegian Ministry of Health and Care Services (2020). National Health and Hospital Plan (2020–2023).

[17] Malterud K, Siersma VD, Guassora AD (2016). Sample size in qualitative interview studies: guided by information power. Qual Health Res.

[18] Eaton K, Stritzke WGK, Ohan JL (2019). Using scribes in qualitative research as an alternative to transcription. Qual Rep.

[19] Norwegian Center for Research Data (2018). Norwegian center for research data.

[20] World Medical Association (2018). WMA Declararions of Helsinki- Ethical principles for medical research involving human subjects.

[21] International QSR (2018). Pty Ltd. NVivo (version 12).

[22] Braun V, Clarke V (2006). Using thematic analysis in psychology. Qual Res in Psychol.

[23] Karlsson AC, Gunningberg L, Bäckström J, Pöder U (2019). Registered nurses’ perspectives of work satisfaction, patient safety and intention to stay – a double-edged sword. J of Nurs Manag.

[24] Kowalczuk K, Krajewska-Kułak E, Sobolewski M (2020). Working excessively and burnout among nurses in the context of sick leaves. Front Psychol.

[25] James-Scotter M, Walker C, Jacobs S (2019). An interprofessional perspective on job satisfaction in the operating room: a review of the literature. J Inteprof Care.

[26] Malley A, Kenner C, Kim T, Blakeney B (2015). The role of the nurse and the preoperative assessment in patient transitions. AORN J.

[27] Ahlstedt C, Eriksson Lindvall C, Holmström IK, Muntlin AÅ (2019). What makes registered nurses remain in work? An ethnographic study. Int J Nurs Stud.

[28] Lögde A, Rudolfsson G, Broberg RR, Rask-Andersen A, Wålinder R, Arakelian E (2018). I am quitting my job. Specialist nurses in perioperative context and their experiences of the process and reasons to quit their job. Int J Qual Health Care.

[29] Rasmussen MB, Tolsgaard MG, Dieckmann P, Østergaard D, White J, Plenge P (2020). Social ties influence teamwork when managing clinical emergencies. BMC Med Educ.

[30] Andersen BR, Hinrich JL, Rasmussen MB, Lehmann S, Ringsted C, Løkkegaard E (2020). Social ties between team members affect patient satisfaction: a data-driven approach to handling complex network analyses. Adv Health Sci Educ Theory Pract.

[31] Halligan M, Zecevic A (2011). Safety culture in healthcare: a review of concepts, dimensions, measures and progress. BMJ Qual Saf.

[32] Harries RL, Bradshaw CA, Jones EA, Lewis P (2013). To admit or not to admit on the morning of surgery patients' perspectives on day of surgery admission. J Perioper Pract.

[33] Svendsen MT, Bak CK, Sørensen K, Pelikan J, Riddersholm SJ, Skals RK (2020). Associations of health literacy with socioeconomic position, health risk behavior, and health status: a large national population-based survey among Danish adults. BMC Public Health.

[34] Prabhakar A, Helander E, Chopra N, Kaye AJ, Urman RD, Kaye AD (2017). Preoperative assessment for ambulatory surgery. Curr Pain and Headache Rep.

[35] Ruhaiyem ME, Alshehri AA, Saade M, Shoabi TA, Zahoor H, Tawfeeq NA (2016). Fear of going under general anesthesia: a cross-sectional study. Saudi J Anaesth.

[36] Ay AA, Ulucanlar H, Ay A, Ozden M (2014). Risk factors for perioperative anxiety in laparoscopic surgery. JSLS.

[37] Bansal T, Joon A (2016). Preoperative anxiety-an important but neglected issue: a narrative review. Indian Anaesth Forum.

[38] Abelsson A, Falk P, Sundberg B, Nygårdh A (2020). Empowerment in the perioperative dialog. Nurs Open.

[39] Schmidt M, Eckardt R, Scholtz K, Neuner B, von Dossow-Hanfstingl V, Sehouli J (2015). Patient empowerment improved perioperative quality of care in cancer patients aged ≥ 65 years - a randomized controlled trial. PLoS One.

[40] European Commission (2019). State of health in the EU, Norway, country Health Profile 2019.

[41] Xiang W, Yin J, Lim G (2015). An ant colony optimization approach for solving an operating room surgery scheduling problem. Comput Ind Eng.

[42] Al Talalwah N, McIltrot KH (2019). Cancellation of surgeries: integrative review. J Perianesth Nurs.

[43] Sanfilippo F, Noto A, Foresta G, Santonocito C, Palumbo GJ, Arcadipane A (2017). Incidence and factors associated with burnout in anesthesiology: a systematic review. Biomed Res Int.

[44] Johnson DR, Scheitle CP, Ecklund EH. Beyond the in-person interview? How interview quality varies across in-person, telephone, and Skype interviews. Soc Sci Comput Rev. 2021;39:1142–58. 089443931989612.

