# Supplementary material for: Knowledge, safety, and teamwork: a qualitative study on the experiences of anaesthesiologists and nurse anaesthetists working in the preanaesthesia assessment clinic
Source: BMC Anesthesiol. 2022 Oct 3;22:309. doi: 10.1186/s12871-022-01852-w (PMC9527137; doi:10.1186/s12871-022-01852-w)
Supplement: Supplementary file 1 — Additional file 1. Examples from the analysis process when generating themes. [file 12871_2022_1852_MOESM1_ESM.docx]

Additional file 1

Examples from the analysis process when generating themes

| Examples of text coded | Subthemes | Themes |
| --- | --- | --- |
| “And I learn a lot as a nurse anaesthetists and pretty much just from sitting in the PAC, because you gain knowledge in a totally different way. And you have a close dialogue with the anaesthesiologist” (participant 2). | Increased learning through the structured assessments of patients | Developing competence in clinical anaesthesia practice |
| “This is what it is like to do interdisciplinary work. Because, I think that's really fun. Ehh ..... there is something about you being forced to work with the other person down there. Ehh .... and it is important there to function socially together, i.e., communicate and listen to each other's arguments around the patient for example” (participant 1). | Internal teamwork | Barriers and facilitators of collaboration and teamwork |
| “But most people think it is ok to talk to somebody in advance. They think it is safe and many have some questions and experiences they want to share” (participant 9). | Improving patient knowledge, experience, and involvement | Improving patient safety and outcomes through a structured assessments |
| “It can be very busy ... ehh ... if there are patients who are sick and unexplained, those require a little more time than the time set aside” (participant 1). | Increase in workload pressure | Organisational factors affecting anaesthesia personnel and delivery of healthcare to surgical patients |

PAC, preanaesthesia assessment clinic
